# Evaluation of the Proliferation Marker Ki-67 for Improved Risk Stratification of Prostate Cancer Patients Under Active Surveillance

**DOI:** 10.3390/diagnostics16070975

**Published:** 2026-03-25

**Authors:** Viktoria Schütz, Maresa Rothermel, Adam Kaczorowski, Svenja Dieffenbacher, Sarah Heike Böning, Constantin Schwab, Albrecht Stenzinger, Johannes Huber, Anette Duensing, Markus Hohenfellner, Stefan Duensing

**Affiliations:** 1 Department of Urology, Heidelberg University Hospital, Im Neuenheimer Feld 420, 69120 Heidelberg, Germanyjohannes.huber@med.uni-heidelberg.de (J.H.);; 2 Molecular Urooncology, Department of Urology, Heidelberg University Hospital, Im Neuenheimer Feld 517, 69120 Heidelberg, Germany; 3 Institute of Pathology, University Hospital Heidelberg, Im Neuenheimer Feld 224, 69120 Heidelberg, Germany; 4 Precision Oncology of Urological Malignancies, Department of Urology, Heidelberg University Hospital, Im Neuenheimer Feld 517, 69120 Heidelberg, Germany

**Keywords:** prostate cancer, active surveillance, risk stratification, tumor marker, Ki-67

## Abstract

**Background/Objectives**: Active surveillance (AS) is a viable option for patients with low-risk/low-burden prostate cancer (PCa). Approximately 40–50% of patients will develop disease progression and conversion to active treatment. Therefore, better risk stratification may aid patients and urologists to improve decision making. Herein, the proliferation marker Ki-67 was examined for its prognostic potential in AS patients. **Methods**: Fifty-nine patients were included. Median follow-up time was 58 months (range, 10–162 months). Tumor-bearing biopsies were evaluated using immunohistochemistry (IHC) staining for Ki-67 and evaluated using digital imaging analysis to determine the percentage of Ki-67-positive PCa cells per biopsy. **Results**: Thirty-three of 59 patients (55.9%) developed progression. Thirty-one of 59 patients (52.5%) showed Ki-67-positive biopsies (median 0.8%; range, 0–11.9%). The median of Ki-67-positive cells was 1.5% (range, 0–11.9%) in patients with and 0% (range, 0–6.3%) in patients without progression. Comparing patients with Ki-67-positive and Ki-67-negative biopsies showed a worse progression free survival (PFS) in patients with Ki-67-positive biopsies after a period of 15 months, however, without reaching statistical significance (*p* = 0.071). A 5% threshold for Ki-67 positivity led to a significant difference in PFS. Further exploratory analysis revealed that patients with Ki-67-positive biopsies and aged ≥65 years or with >1 tumor-bearing biopsy show a significantly worse outcome (*p* = 0.038 and *p* = 0.037, respectively). **Conclusions**: Our results suggest that patients with Ki-67-positive biopsies remaining in AS for >1 year have an increased risk for PCa progression and conversion to treatment. Studies to further confirm Ki-67 as a marker for risk stratification, especially with a positivity cut-off of 5%, are warranted in larger cohorts of AS patients.

## 1. Introduction

Prostate cancer (PCa) is the most common non-cutaneous cancer in men [1] and often detected in an organ-confined, curable stage, especially in countries where PSA-based screening is available [2,3]. For men with low-risk/low-burden PCa detected by a multiparametric (mp) MRI-guided prostate biopsy, an active surveillance (AS) strategy can be a viable option to avoid overtreatment [4,5,6,7] and to delay possible side effects of active treatment by radical prostatectomy (RP) or radiation therapy (RT) [6]. Patients in AS undergo regular clinical examinations including digital rectal examination (DRE), PSA-testing and repeat prostate biopsies [6]. Repeat biopsies should be performed after the first year and then once every three years over a period of ten years [6]; however, repeat biopsies may be omitted when PSA and imaging results are stable [8]. Whether mpMRI alone is suitable for follow-up of AS patients is currently under investigation [9]. Factors that trigger an earlier follow-up biopsy are a rising PSA level or clinical progression on DRE or mpMRI [8,10]. Conversion to active treatment should not occur without a confirmatory biopsy [10] showing an upgrade in histopathology and/or to stage ≥ cT2c [10,11].

Disease progression and, therefore, the need for active treatment is seen in approximately 20–40% of patients under AS [5,12,13,14]. With more intermediate-risk PCa patients being included in AS there is a need for further risk stratification. The ProtecT trial has shown that there was no difference in prostate-cancer-specific death over a 15-year follow-up period regardless if patients underwent a monitoring strategy or active treatment (RP or RT) [15]. It needs to be pointed out that patients in the monitoring group did not undergo a regular follow-up in accordance with AS protocols. Patients under monitoring appear to be at a higher risk of developing metastatic disease (9.4% for men under AS vs. 4.7% after RP and 5.0% after RT) and clinical progression (25.9% for men under AS vs. 10.5% after RP and 11% after RT) [15]. This can be an indicator that patients eligible for AS or a monitoring strategy should undergo further risk stratification.

In the light of these findings, the development of novel biomarkers that predict disease progression and the need for a conversion to active treatment are still needed to improve decision making for patients and urologists. Different approaches to develop additional prognostic tools have been made. For example, various clinical parameters have been identified for prostate cancer risk stratification such as PSA level, the percentage of free PSA [16], PSA density [13,16], the maximum percentage of core involvement per biopsy core [16] or the number of positive cores [13,17]. Additional factors such as age [18], race [13,19] or a positive family history [20] have been discussed. Together with PSA level, Grade Group and prostate volume, mpMRI scores have been used to develop a model for predicting the risk of progression for men starting AS [21]. In addition, *BRCA1/2* mutations are associated with an adverse outcome and a more aggressive course of disease in PCa patients [22,23,24]. Changes in the p53 protein [25,26] and a loss of *PTEN* [27,28,29] are likewise associated with more aggressive forms of PCa and poor clinical outcomes. In fact, loss of *PTEN* has been reported as a marker for exclusion of patients from AS [30].

Another marker that has been investigated in PCa patients is the proliferation marker Ki-67. Ki-67 has been found to be associated with an adverse outcome in prostate cancer patients [31,32,33,34,35,36,37,38,39,40] including a higher pathological stage, a higher Grade Group, seminal vesical invasion and a significantly worse recurrence free survival [31,37,41]. Evaluations of Ki-67 as a predictive marker in AS are limited [35,42,43,44].

Herein, we investigate Ki-67 and its prognostic value in prostate cancer patients under AS. We also evaluate Ki-67 status in combination with other clinic-pathological parameters to improve risk stratification in AS patients.

## 2. Patients and Methods

### 2.1. Patient Population

A total of 59 patients were included in this single-center analysis. All patients had biopsy-proven low-risk PCa diagnosed between 2006 and 2017. Patients were classified as low-risk in accordance with D’Amico criteria [45] in line with published literature [46]. Forty-five of 59 patients (76.3%) underwent a mpMRI-guided biopsy. A subset of patients were diagnosed before mpMRI of the prostate became widely available and were therefore staged by digital rectal examination in accordance with the AJCC classification from 2002 [47]. Tissue samples for Ki-67 immunohistochemistry (IHC) were available for all 59 patients. Fourteen patients (23.7%) had received a previous biopsy and their first diagnosis elsewhere. The median follow-up time was 58 months (range, 10–162 months). Patients were included consecutively based on their eligibility for AS. At the time of diagnosis, all patients met the criteria for AS. According to national guidelines at the time of inclusion, patients received regular clinical follow-ups including PSA measurements, imaging as well as repeat prostate biopsies at defined time intervals [48]. Clinical information was retrieved from the hospital information system of Heidelberg University Hospital and from the tumor database of the Department of Urology of Heidelberg University Hospital, which is a prospectively run data base collecting data from uro-oncological patients. Progression was defined by histological upgrade on repeat biopsy, PSA-progression, clinical progression or a combination of the mentioned parameters. Patients gave informed written consent for the use of their data and tissue for research and publication. Tissue samples were stored at and provided by the tissue bank of the National Center for Tumor Diseases (NCT), Heidelberg, Germany, in accordance with regulations of the tissue bank and the approval of the ethics committee of the Medical Faculty Heidelberg of the University of Heidelberg (206/2005, 207/2005, S-864/2019, S-287/2022).

### 2.2. Tissue Selection

Only tissue sections from FFPE blocks with histopathologically confirmed tumor infiltration were retrieved from the NCT tissue bank. All sections were microscopically re-examined using H&E staining and the sample (=FFPE block) containing the maximum amount of viable tumor was selected for further examination. The samples were then stained for Ki-67 by immunohistochemistry. Digital images were captured only from samples containing Ki-67-positive tumor cells and transferred to QuPath [45]. All others were classified as negative without further QuPath analysis. While the maximum amount of tumor cells was used for sample selection (=FFPE block), all additional tumor containing areas were included in the QuPath analysis and the ratio of Ki-67-positive tumor cells to the overall number of tumor cells was calculated. Tissue samples were regarded as Ki-67-negative if there were no proliferating cells detectable in the areas analyzed and no Ki-67-positive cells were detected (“0%”). If the ratio of Ki-67-positive tumor cells to tumor cells overall was >0%, the tissue sample was regarded as Ki-67-positive. All QuPath scans were independently analyzed by two observers, no blinded analysis was performed.

### 2.3. Immunohistochemistry

Biopsy tissue was analyzed for Ki-67-positive PCa cells using IHC. Formalin-fixed, paraffin-embedded (FFPE) tissue sections from biopsies were deparaffinized by immersion in xylol for a total of 12 min. The slides were then rehydrated in a graded series of ethanol. Antigen retrieval was performed with Target Retrieval Solution (Dako, Glostrup, Denmark) for 35 min. Peroxidase quenching was performed using 3% H_2_O_2_ in methanol for 10 min. To block non-specific antibody binding, 10% normal goat serum (Dako) was applied for 30 min. Slides were then incubated with the primary antibody at 4 °C overnight. A monoclonal mouse anti-human Ki-67 antigen clone MIB-1 was used (Agilent Technologies, Inc., Santa Clara, CA, USA, ready-to-use). The slides were incubated afterwards with a secondary biotin-conjugated antibody (goat anti-mouse IgG H&L, Invitrogen by Thermo Fisher Scientific, Waltham, MA, USA, 1:200) at 37 °C for three hours and incubated with a streptavidin-POD-conjugate (Roche Diagnostics, GmbH, Rotkreuz, Switzerland) diluted in PBS (1:1250) at room temperature for 30 min. Afterwards the slides were washed in PBS. Slides were then stained with a DAB solution (DAB Substrate Kit, Abcam, ab64238, Cambridge, UK). Counterstaining was performed with hematoxylin for 5 sec (Hematoxylin Solution, Gill No.1, Sigma-Aldrich, St. Louis, MO, USA). The tissue was then dehydrated and mounted using Histomount (Invitrogen, Waltham, MA, USA).

As a positive control, an array containing four FFPE embedded PCa cell lines (LNCaP, PC-3, 22Rv1, DU-145; AMSBIO, Abingdon, UK) was used in every experiment. Only experiments with a positive staining of these four cell lines were included in the analysis. The stained slides were examined using a Leica DM750 microscope (Leica, Wetzlar, Germany) equipped with a 10× objective and a 10× eyepiece leading to a field of view of 0.785 mm^2^. Results were documented using a Leica DM5000 B microscope equipped with a K3C camera (Leica). Pictures with positive results were analyzed using QuPath (Version 0.5.1) for digital image analysis to quantify the percentage of Ki-67-positive PCa cells.

### 2.4. Statistical Analysis

*p*-values were calculated using the Mann–Whitney U test. Statistical significance was set at *p* < 0.05. The Kaplan–Meier method with log-rank statistics was used. Descriptive analysis was performed using R Studio (Version 2024.04.1+748) and Microsoft Excel (Version 2411). Statistical analysis was conducted using IBM SPSS Statistics for Windows, Version 27 (IBM Corp., Armonk, NY, USA).

## 3. Results

### 3.1. Clinico-Pathological Characteristics of the AS Cohort

A total of 59 patients with low-risk PCa under AS were included and analyzed for Ki-67 positivity. A median number of 24 biopsies were taken per patient (range, 8–40). The median follow-up time after start of AS was 58 months (range, 10–162 months). The presence of low-risk PCa with a Gleason Score of 6 was confirmed by in-house prostate biopsy. Basic patient characteristics are shown in Table 1.

Disease progression and conversion to active treatment was observed in 33 patients (55.9%). Median time to progression was 21 months (range, 6–148 months). The main cause for disease progression and end of AS was histological upgrading to a more aggressive histology in repeat biopsies (*n* = 23, 69.7%). Other causes included radiological progression (*n* = 3, 9.2%), clinical progression (*n* = 1, 3.0%), PSA-progression (*n* = 2, 6.1%) or a combination of the above (*n* = 4, 14.8%; Table 2). Patients with disease progression received a radical prostatectomy (RP; *n* = 16, 48.5%), radiotherapy (RT; *n* = 1, 3.0%) or other treatment. Four patients (6.8%) decided to leave AS and switch to active treatment without signs of disease progression (Table 2).

In patients undergoing active treatment by RP, locally advanced disease (pT3) was observed in five patients (31.3%), while 11 patients (68.8%) had organ-confined disease (pT2, Table 3). In one patient (6.3%), lymph node metastases were present at the time of RP. During follow-up, eight patients (24.2%) developed biochemical recurrence (BCR) after a median time interval of 10 months (range, 2–29 months, Table 3).

There were no statistically significant differences with respect to clinico-pathological parameters in patients with and without disease progression analyzed (Table 4).

### 3.2. Ki-67 Immunohistochemistry of Prostate Biopsies Correlates with Patient Survival in an Exploratory Analysis

**Figure 1:**
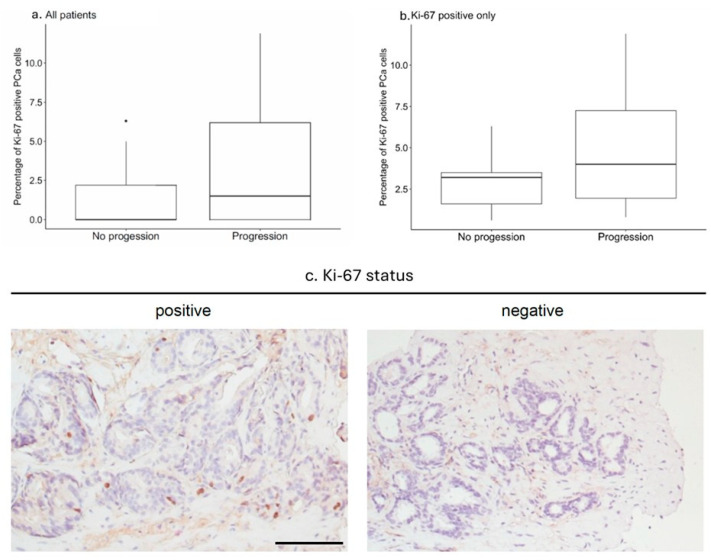
Ki-67-positive PCa cells in patients in AS. Percentage of Ki-67-positive PCa cells in AS patients with (*n* = 33, 60%) or without (*n* = 22, 40%) disease progression (*p* = 0.081) (**a**). A further analysis of patients with Ki-67-positive biopsies (*n* = 29) revealed in patients with disease progression (*n* = 20) a Ki-67 positivity rate of 39.0% in comparison to 31.0% in patients without disease progression (*n* = 9). This difference did not reach statistical significance (*p* = 0.248) (**b**). Bar graphs indicate median, interquartile range and minimum and maximum; points indicate outliers. Ki-67-positive and negative IHC staining in PCa patients in AS. Representative microphotographs of Ki-67-positive (proliferation index = 6.2%) and negative tumor tissue are shown. Scale bar = 20 µm (**c**).

IHC staining for Ki-67 was performed as described above. An example of the staining results is shown in Figure 1c. In 31 of 59 patients (52.5%), biopsies were found to contain Ki-67-positive PCa cells.

Disease progression was observed in 33 patients (55.9%). In these patients, the median percentage of Ki-67-positive tumor cells was 1.5% (range, 0–11.9%) when compared to patients without progression, which showed a median of 0% positive tumor cells (range, 0–6.3%; Figure 1a).

Patients with Ki-67 positively stained biopsies (*n* = 29) were further evaluated. Patients with a disease progression (*n* = 20) showed a median percentage of Ki-67-positive tumor cells of 4% (range, 0.8–11.9%). While those with no disease progression (*n* = 9) had a median of 3.2% of Ki-67-positive tumor cells (range, 0.6–6.3%) as shown in Figure 1b.

For the survival analysis, patients were stratified according to the presence (i.e., one or ≥1) or absence of Ki-67-positive PCa cells in their biopsies. In accordance with the literature [49,50], progression free survival (PFS) was also analyzed using 1% and 5% Ki-67 positivity as cut-off values for further risk stratification. Since a proportion of patients were first diagnosed outside the Department of Urology Heidelberg, we decided to use two time intervals for the survival analysis. First, from the time of first diagnosis to disease progression and second, from the time from in-house biopsy to disease progression.

**Figure 2:**
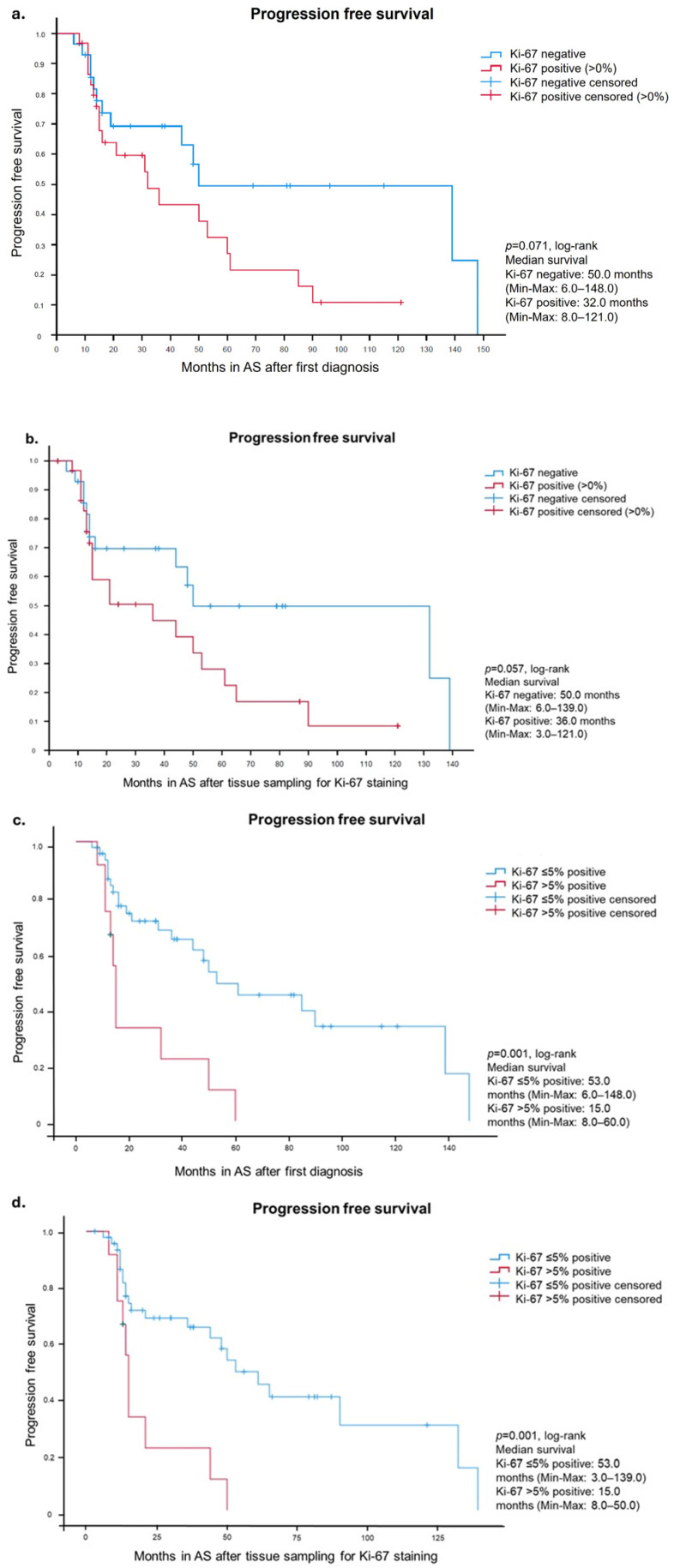
Worse progression free survival in patients with Ki-67-positive PCa cells in prostate biopsy. Kaplan–Meier curves showing months in AS in patients with and without Ki-67-positive PCa cells starting at time of diagnosis (**a**). Worse progression free survival in patients with Ki-67-positive PCa cells in prostate biopsies. Kaplan–Meier curves showing months in AS in patients with and without Ki-67-positive PCa cells starting at time of tissue sampling (**b**). Significantly worse progression free survival in patients with more than 5% Ki-67-positive tumor cells in prostate biopsy. Kaplan–Meier curves showing months in AS in patients with ≤5% and >5% Ki-67-positive PCa cells starting at the time of diagnosis (**c**). Significantly worse progression free survival in patients with more than 5% Ki-67-positive tumor cells in prostate biopsy. Kaplan–Meier curves showing months in AS in patients with ≤5% and >5% Ki-67-positive PCa cells starting at the time of in-house tissue sampling (**d**).

Differences in PFS from the time of diagnosis were observed between patients with and without Ki-67-positive biopsies. During the first approximately thirteen months after diagnosis, the survival curves for patients with Ki-67-positive and negative biopsies were similar. However, over time, a separation of the two curves was observed with patients having Ki-67-positive biopsies showing a more rapid disease progression (Figure 2a). Median PFS for patients with Ki-67-positive biopsies was 32 months (range, 8–121 months), compared to 50 months (range, 6–148 months) for patients with Ki-67-negative biopsies. Our results therefore suggest a less favorable PFS in patients with Ki-67-positive biopsies, although not reaching statistical significance (*p* = 0.071).

For a second Kaplan–Meier analysis, the PFS was calculated starting from the time of in-house biopsy. Again, the Kaplan–Meier curves are similar in the beginning but start separating over time, favoring patients with Ki-67-negative biopsies (Figure 2b). Median PFS after tissue sampling for patients with Ki-67-positive biopsies was 36 months (range, 3–121 months), compared to 50 months (range, 6–139 months) in patients with Ki-67-negative biopsies. A more favorable PFS was also evident when the in-house biopsy procedure was used as the starting point, although it did not reach statistical significance (*p* = 0.057).

Further analyses were calculated using 1% and 5% Ki-67 positivity as thresholds. A 1% Ki-67 positivity cut-off did not show a significant difference in the PFS between patients with less or more than 1% Ki-67 positivity. However, a threshold of 5% revealed a significant difference in patients with >5% Ki-67-positive tumor cells compared to patients with ≤5% Ki-67-positive tumor cells. Median PFS after diagnosis (Figure 2c) for patients with >5% Ki-67-positive tumors cells was 15.0 months (range, 8–60 months), compared to 53 months (range, 6–148 months) in patients with ≤5% Ki-67-positive tumor cells (*p* = 0.001). Using the in-house biopsy procedure as the starting point (Figure 2d) revealed the same results with a significantly better PFS in patients with <5% positive tumor cells (*p* = 0.001).

For further evaluation of the effect of Ki-67 status, a uni- and multivariate Cox regression analysis was performed for both of the above-described time intervals. Due to the fact that pathology reporting standards changed over time, we decided to only include patients in multivariate Cox regression analysis for which pathology reporting met standardized criteria. Therefore, the patient numbers for multivariate Cox regression analysis decreased to 32. Although not reaching statistical significance, patients with Ki-67-positive biopsies were at a higher risk of experiencing disease progression than patients with Ki-67-negative biopsies (Supplementary Table S1). The hazard ratio for disease progression starting at the time of diagnosis was 2.026 (95% CI 0.975–4.211) in patients with Ki-67-positive biopsies (*p* = 0.059). A similar result was observed regarding PFS starting from the time of in-house tissue sampling with a hazard ratio of 2.081 (95% CI 1.001–4.324); (*p* = 0.050). In multivariate Cox regression analysis starting at the time of diagnosis patients with Ki-67-negative biopsies had a hazard ratio of 0.710 (95% CI 0.205–2.464) for disease progression in comparison to patients with Ki-67-positive biopsies without reaching statistical significance (*p* = 0.590). Statistical significance was also not reached when the time of in-house tissue sampling was used as the starting point (Supplementary Table S2).

These analyses were repeated using a Ki-67 positivity threshold of 5% (Supplementary Table S1). For both time intervals, starting from the time of first diagnosis and from the time of in-house tissue sampling, patients with more than 5% Ki-67-positive tumor cells showed a significantly higher risk of disease progression than patients with ≤5% Ki-67-positive tumor cells (*p* = 0.004, respectively). Although Ki-67 positivity of 5% was a significant predictor of disease progression, it was not significant in multivariate Cox regression analysis (Supplementary Table S3). However, because of the limited number of patients our multivariate analysis should be considered exploratory.

### 3.3. Additional Clinical Parameters May Enhance Diagnostic Accuracy of Ki-67

**Figure 3:**
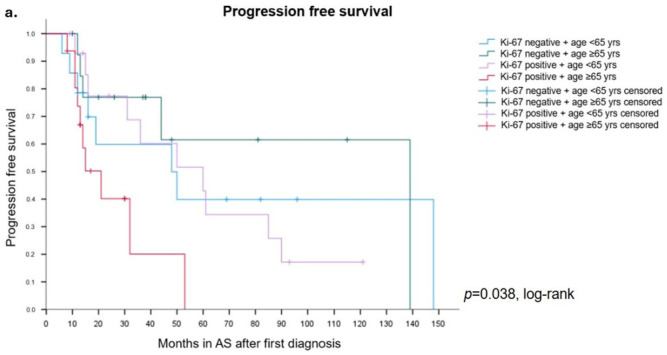

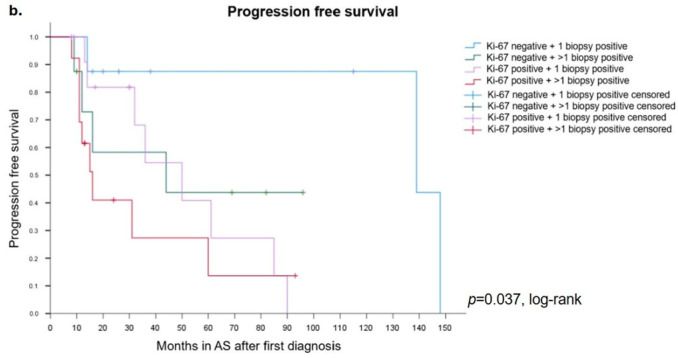
Progression free survival comparing patients aged <65 or ≥65 years and with Ki-67-positive or negative prostate biopsies. The worst outcome was observed for patients with Ki-67-positive biopsies aged ≥65 years. Kaplan–Meier curves showing time in AS after initial diagnosis (**a**). Progression free survival comparing patients with one or more than one positive biopsy and positive or negative Ki-67 expression. The worst outcome was observed for patients with Ki-67-positive biopsies and more than one positive biopsy. Kaplan–Meier curves showing time in AS after initial diagnosis (**b**).

Because the Ki-67 risk stratification of biopsies into positive and negative is straight forward, we next attempted to increase prognostic accuracy by including additional variables (Supplementary Table S4). Of these parameters, Ki-67 status in combination with the number of tumor-bearing biopsies (1 vs. >1 tumor-bearing biopsy) and the patient age at time of diagnosis (≥65 vs. <65 years) had significant influences on PFS (log rank, *p* = 0.037 and *p* = 0.038, respectively) starting from the time of diagnosis. Results are shown in Supplementary Table S1 and in Figure 3a,b. Together with age and the number of tumor-bearing biopsies, Ki-67 can significantly improve risk stratification in PCa patients under AS.

## 4. Discussion

Active surveillance is a viable option for patients with low-risk/low-burden PCa to avoid overtreatment and delay possible side effects of RP or RT. However, a conversion to active treatment is seen in up to 40% of patients under AS within five years [5,12,13,14,51]. Different clinical parameters and biomarkers have been evaluated for the risk stratification of PCa patients under AS. In this retrospective, single-center study we analyzed the proliferation marker Ki-67 and its prognostic value in PCa patients under AS.

We found that patients with disease progression not only had a higher median percentage of Ki-67-positive tumor cells, but also that the presence of a single Ki-67-positive biopsy was associated with a worse PFS. Furthermore, a threshold of more than 5% Ki-67 positivity correlated with a significantly worse PFS. These differences in patient survival became obvious after approximately one year of AS. Further explorative data analyses revealed that patients with Ki-67-positive biopsies showed a significantly worse survival when aged ≥65 years or had more than one tumor-bearing biopsy. Ki-67 may therefore be a possible predictive marker for low-risk/low-burden PCa patients who stay under AS for more than one year and can also predict a significantly worse outcome in patients aged ≥65 years or patients with more than one tumor-bearing biopsy. Further studies with a larger patient cohort are needed to confirm these results from our exploratory and hypothesis-generating analysis.

The proliferation marker Ki-67 was first described in the 1980s [52]. Our results suggesting a more unfavorable prognosis associated with Ki-67 positivity, and in particular with a Ki-67 positivity threshold of greater 5%, are well in line with numerous studies showing the association in cancer in general [53,54,55], as well as in PCa [31,32,33,34,36,40,42,43,44].

Studies analyzing Ki-67 in prostate biopsies from patients in AS are limited. A previous study by Jhavar et al. reports a median Ki-67 labeling index of 1 with a range of 0 to 8.75 [35], which is very similar to the median of 0.8% and the range of 0% to 11.9% in our study. Kammerer-Jacquet et al. used a cut-off value of 5% for Ki-67 in prostate biopsies in patients with conservatively managed prostate cancer to predict disease specific mortality [49]. However, the cohort composition was vastly different than in our study with 76% of patients having a Grade Group of 2 or higher [49]. Only 24% showed Grade Group 1 and hence potentially qualified for AS [49]. Since the Ki-67 positivity increases with higher grading, the cut-off of 5% in this unselected biopsy patient cohort is not surprising. In line with this notion, we found that a cut-off threshold for Ki-67 positivity of 5% predicts drop-out from AS.

When comparing Ki-67 positivity in prostate biopsies to results from the analysis of prostatectomy specimens, one should keep in mind that in biopsies only a small fraction of the tumor is analyzed, therefore prostate biopsies will always and intrinsically have a sampling bias. Poor tissue fixation, inadequate antigen retrieval and edge-effects are unlikely to have affected our results because biopsies are routinely transferred directly into fixation medium and, because of their small size, are immediately fixed in toto. Nevertheless, we strongly recommend the use of a positive control as described in our Patients and Methods section to rule out false negative results.

Our results furthermore suggest that Ki-67 status, together with age at diagnosis and the number of tumor-bearing biopsies, influences PFS. In our cohort, we found an overall progression rate of 55.9% over a follow-up period of more than ten years, which is in accordance with progression rates of 40–50% reported in the literature [12,56]. Our findings confirm and extend previous studies showing that the number of tumor-bearing biopsies [14,57,58] and patient age are risk factors for disease progression [57,59,60,61], although Ki-67 was not part of these analyses. While the number of tumor-bearing biopsies is clearly a reflection of tumor burden, the role of patient age was somewhat surprising. Advanced age at diagnosis has been described to be associated with a poorer outcome [60,62,63]. Moreover, it has been reported that a younger AS patient age (≤60 years) is linked to a lower-risk of Gleason Score/Grade Group upgrade on repeat biopsy [64].

Despite the small sample size, our results have translational relevance, in particular in the light of the emerging trend to include patients with Grade Group 2 into AS. For example, in the ProtecT trial, no difference in cancer-specific mortality was reported over a 15-year follow-up period. However, patients under observation still developed metastases, clinical progression and needed systemic treatment more frequently than patients receiving active treatment [15]. Hamdy et al. discuss that conventional criteria such as PSA level, clinical stage and Gleason Score/Grade Group might therefore not be sufficient for risk stratification and treatment in intermediate-risk PCa patients [15]. In the PIVOT trial the authors reported similar results. 731 men with localized PCa were randomized either into an observation (n = 367) or a RP (n = 364) group [65]. No differences were observed in overall and PCa-specific mortality after a follow-up time of 12 to 19.5 years [65], including their long-term follow-up evaluation [66]. However, fewer men assigned to RP experienced disease progression (40.9% vs. 68.4%) and a need for treatment due to disease progression (33.5% vs. 59.7%) [65]. The authors therefore suggested that strategies to identify patients benefitting from an early treatment while avoiding overtreatment are needed [66].

It is important to mention that neither the ProtecT nor the PIVOT trial followed current AS protocols and did not require mpMRI of the prostate at initial diagnosis or during follow-up [15,65]. However, evidence on prostate mpMRI fundamentally changing the management of AS patients is mounting [67,68,69]. Guidelines recommend mpMRI-guided biopsy for PCa patients before enrolling in AS [6]. Current studies have also evaluated the role of mpMRI for follow-up in AS patients to possibly avoid repeat-biopsies [9] and standard criteria have been defined to assess PCa progression on mpMRI [70]. Although, data does not yet support a surveillance strategy based on mpMRI alone [71,72,73]. Therefore, further strategies to improve risk stratification in AS patients beyond current criteria are required.

As suggested by our results and supported by results from previous studies, Ki-67, together with other factors such as the number of tumor-bearing biopsy cores or patient age in combination with conventional factors (e.g., PSA, PSA-density) and prostate mpMRI, might be a possible strategy to identify patients at risk of disease progression and improve risk stratification in patients eligible for AS. Other molecular markers that may help to aid risk stratification in AS patients are mutations in *TP53*, a loss of *PTEN* or *BRCA1/2* mutations indicating an aggressive course of disease [23,24,26,27,30,74,75]. Whether patients with *BRCA1/2* mutations should undergo AS is still under debate [6]. A positive family history could trigger testing for a *BRCA2* mutation and might influence AS decision making [6]. Additional markers such as *TP53* and *PTEN* could, in combination with Ki-67, further support our findings and enhance prognostic accuracy.

The limitations of this study are the overall limited number of patients, the fact that 18 patients were lost to follow-up and the single center, retrospective character. These aspects should be kept in mind when interpreting the results as a larger cohort would be needed to confirm our findings. Moreover, some patients were first diagnosed at outside institutions. Our study also did not include Grade Group 2 patients. Especially in the light of recently changed guidelines, this patient group and their outcome under AS should be further studied. In comparison to other cohorts, our results clearly show a lower Ki-67 expression. However, we report on a highly selected patient cohort only containing low-risk/low-burden PCa patients, while most previous studies mainly included intermediate- and high PCa patients. Despite the limited patient number, our analysis is still relevant and serves as a hypothesis-generating, proof-of-concept study. Our results could provide the basis for future studies prospectively evaluating Ki-67, in particular with a Ki-67 positivity threshold of 5%, or a combination of markers including Ki-67 to improve risk stratification in low-risk/low-burden prostate cancer patients eligible for active surveillance.

To further evaluate the prognostic impact of Ki-67 in AS patients, prospective, long-term and ideally multicenter studies would be needed with specific protocols regarding tissue-sampling and follow-up. Comparing Ki-67 status between prostate biopsy and prostatectomy specimens in patients with disease progression undergoing RP could add further value to Ki-67 as a predictive marker.

## 5. Conclusions

Our results suggest that Ki-67 is a possible marker for risk stratification in patients eligible for AS. Together with patient age (≥65 years) and the number of tumor-bearing biopsies (*n* > 1), patients with Ki-67-positive biopsies show a significantly worse survival compared to patients without these features. Patients with positive biopsies and a Ki-67 positivity rate of >5% show a significantly worse PFS compared to patients with ≤5% Ki-67 positivity. To further validate our results, larger patient cohorts and possibly prospective and multicenter studies are warranted.

## Figures and Tables

**Table 1:** Patient characteristics at start of active surveillance (*n* = 59).

Variable	
Age, years, median (IQR)	65 (59.5–68)
PSA, ng/mL, median (IQR)	5.7 (4.5–7.2)
Prostate volume, mL, median (IQR)	39 (31.5–56.9)
PSA density, ng/mL/cc, median (IQR)	0.12 (0.09–0.18)
Digital rectal examination (DRE) suspicious, *n* (%)	
Yes	8 (13.6)
No	50 (84.7)
Unknown	1 (1.7)
mpMRI, *n* (%)	
Yes	45 (76.3)
No	6 (10.2)
Unknown	8 (13.6)
Clinical T Stage, *n* (%)	
cT1	51 (86.4)
cT2	8 (13.6)
Number of biopsies, median (IQR)	24 (23–28)
Unknown, *n* (%)	4 (6.8)
Number of biopsy cores with PCa, median (IQR)	2 (1–2.8)
Unknown, *n* (%)	21 (35.6)
Gleason Score 6, *n* (%)	59 (100)
Maximum PCa infiltration rate, %, median (IQR)	5 (5–10)
Unknown, *n* (%)	6 (10.2)
Follow up time since start of AS, months, median (IQR)	58 (18–95.5)

IQR = interquartile range, PSA = prostate specific antigen, mpMRI = multiparametric MRI, AS = active surveillance.

**Table 2:** Follow-up after active surveillance and treatment for progressive disease (*n* = 59).

Variable	
Overall time on AS, months, median (IQR)	21 (13–50)
Still on AS, *n* (%)	4 (6.8)
Progression, *n* (%)	33 (55.9)
Evidence of progression, *n* (%)	
Histology	23 (69.7)
Radiological	3 (9.1)
Clinical	1 (3.0)
PSA	2 (6.1)
Combination of the above	4 (14.8)
Progression treatment, *n* (%)	
RP	16 (48.5)
RT	1 (3.0)
Other	6 (18.2)
Unknown	10 (30.3)
Conversion to active treatment w/o evidence of progression, *n* (%)	4 (6.8)
Lost to follow-up, *n* (%)	18 (30.5)
Death from other causes, *n* (%)	2 (3.4)

RP = radical prostatectomy, RT = radiation therapy, IQR = interquartile range, AS = active surveillance, PSA = prostate specific antigen.

**Table 3:** Active therapy and outcome (*n* = 33).

Variable	
RP, *n* (%)	16 (48.5)
Pathological T stage pT, *n* (%)	
1c	0 (0.0)
2a	2 (12.5)
2b	0 (0.0)
2c	9 (56.3)
3a	1 (6.3)
3b	4 (25.0)
Pathological N stage, pN, *n* (%)	
N0	13 (81.3)
N1	1 (6.3)
NX	2 (12.5)
Gleason Score/Grade Group, *n* (%)	
6 (1)	2 (12.5)
7a (2)	11 (68.8)
7b (3)	2 (12.5)
8 (4)	0 (0.0)
9 (5)	1 (6.3)
Resection margins, *n* (%)	
R0	14 (87.5)
R1	2 (12.5)
RX	0 (0.0)
RT, *n* (%)	1 (3.0)
Others, *n* (%)	6 (18.2)
Unknown, *n* (%)	10 (30.3)
BCR, *n* (%)	
Yes	8 (24.2)
No	16 (48.5)
Unknown	9 (27.3)
PSA at BCR, ng/mL, median (IQR)	0.49 (0.25–4.9)
BCR-free survival, months, median (IQR)	10 (5–13.5)
Radiological Progression, *n* (%)	
Yes	2 (6.1)
No	19 (57.8)
Unknown	12 (36.4)

RP = radical prostatectomy, RT = radiation therapy, BCR = biochemical recurrence, IQR = interquartile range, PSA = prostate specific antigen.

**Table 4:** Patient characteristics stratified into progression vs. no progression (*n* = 59).

Variable	Progression (*n* = 33)	No Progression (*n* = 26)	*p*
Year of PCa diagnosis, median (range)	2013 (2009–2017)	2013 (2006–2016)	0.299
Age at start of AS, years, median (IQR)	63 (59–69)	65 (60.3–68)	0.884
PSA at start of AS, ng/mL, median (IQR)	5.8 (4.6–7.2)	5.7 (4.7–7.2)	0.647
Prostate volume, mL, median (IQR)	38.0 (31–56)	40.8 (33.3–56.9)	0.658
PSA density, PSA/mL, median (IQR)	0.12 (0.09–0.18)	0.11 (0.09–0.16)	0.497
DRE suspicious, *n* (%)			0.135
Yes	2 (6.1)	6 (23.1)	
No	30 (90.9)	20 (76.9)	
Unknown	1 (3.0)	0 (0.0)	
mpMRI at start of AS, *n* (%)			0.609
Yes	25 (75.8)	20 (76.9)	
No	4 (12.1)	2 (7.7)	
Unknown	4 (12.1)	4 (15.4)	
Clinical T Stage, *n* (%)			0.135
cT1	2 (6.1)	6 (23.1)	
cT2	30 (90.9)	20 (76.9)	
Unknown	1 (3.0)	0	
Number of biopsies, median (IQR)	24 (22–28)	25 (23.8–28)	0.673
Unknown, *n* (%)	2 (6.0)	2 (7.7)	
Number of biopsy cores with PCa, median (IQR)	2 (1–3)	1.5 (1–2)	0.215
Unknown, *n* (%)	10 (30.3)	10 (38.5)	
Maximum PCa infiltration rate, %, median (IQR)	5 (5–15)	5 (5–10)	0.892
Unknown, *n* (%)	1 (3.0)	5 (19.2)	
Follow up time since start of AS, months, median (IQR)	78 (24–108)	33.5 (16.3–80.5)	0.054
Overall time in AS, months, median (IQR)	16 (12–50)	28 (14–63.8)	0.384
Ki-67-positive PCa cells, median (IQR)	1.5 (0–11.9)	0.0 (0.0–6.3)	0.081

PCa = prostate cancer, AS = active surveillance, PSA = prostate specific antigen, mpMRI = multiparametric MRI, IQR = interquartile range, DRE = digital rectal examination.

## Data Availability

The original contributions presented in this study are included in the article. Further inquiries can be directed to the corresponding author.

## Supplementary Materials

The following supporting information can be downloaded at: https://www.mdpi.com/article/10.3390/diagnostics16070975/s1, Table S1: Univariate Cox regression analysis of patients in IHC cohort; Table S2: Multivariate Cox regression analysis of patients in IHC cohort including a Ki-67 positivity (yes vs. no); Table S3: Multivariate Cox regression analysis of patients in IHC cohort including a cut-off Ki-67 positivity rate of 5%; Table S4: Log-rank survival analysis of different models combing Ki-67 status with clinic-pathological parameters.

## Abbreviations

The following abbreviations are used in this manuscript:

AS	Active surveillance
PCa	Prostate cancer
IHC	Immunohistochemistry
RP	Radical prostatectomy
RT	Radiotherapy
DRE	Digital rectal examination
IQR	Interquartile range
PFS	Progression free survival
PSA	Prostate specific antigen
