# Supplementary material for: Evaluation of the Proliferation Marker Ki-67 for Improved Risk Stratification of Prostate Cancer Patients Under Active Surveillance
Source: Diagnostics (Basel). 2026 Mar 25;16(7):975. doi: 10.3390/diagnostics16070975 (PMC13072387; doi:10.3390/diagnostics16070975)
Supplement: Supplementary file 1 [file diagnostics-16-00975-s001.zip › diagnostics-4136146-supplementary.pdf]

Supplementary Table S1. Univariate Cox regression analysis of patients in IHC cohort.

Time interval first diagnosis until progression

Variable	n	Hazard ratio	95% confidence interval	p
Age: < 65 vs. ≥ 65 years*	59	1.170	0.581-2.355	0.661
PSA: <4 vs. ≥4 ng/ml*	59	1.018	0.861-1.203	0.835
Clinical Stage: cT1c vs. cT2*/**	58	1.145	1.047-1.253	0.003
Number of biopsy cores with PCa: 1 vs. >1*	38	2.110	0.890-5.002	0.090
Maximum PCa infiltration rate: <10 vs. ≥10 %**	52	1.648	0.827-3.286	0.156
Ki-67 positive PCa cells: yes vs. no**	59	2.026	0.975-4.211	0.059
Ki-67 positive PCa cells: >5% vs. ≤5%**	59	3.190	1.464-6.951	0.004

* Information from the time of initial diagnosis
** Information from the time of in-house tissue sampling

Time interval in-house tissue sampling until progression

Variable	n	Hazard ratio	95% confidence interval	p
Age: < 65 vs. ≥ 65 years*	59	1.142	0.566-2.301	0.711
PSA: <4 vs. ≥4 ng/ml*	59	1.002	0.847-1.184	0.983
Clinical Stage: cT1c vs. cT2*/**	58	1.118	1.036-1-207	0.004
Number of biopsy cores with PCa: 1 vs. >1*	38	2.208	0.910-5.356	0.080
Maximum PCa infiltration rate: <10 vs. ≥10 %**	53	1.893	0.948-3.780	0.071
Ki-67 positive PCa cells: yes vs. no**	59	2.081	1.001-4.324	0.050
Ki-67 positive PCa cells: >5% vs. ≤5%**	59	3.200	1.454-7.041	0.004

* Information from the time of initial diagnosis
** Information from the time of in-house tissue sampling

Supplementary Table S2. Multivariate Cox regression analysis of patients in IHC cohort including a Ki-67 positivity (yes vs. no).

Time interval first diagnosis until progression

Variable	n	Hazard ratio	95% confidence interval	p
Age: < 65 vs. ≥ 65 years*	32	2.459	0.812-7.447	0.112
PSA: <4 vs. ≥4 ng/ml*	32	1.037	0.817-1.316	0.765
Clinical Stage: cT1c vs. cT2*/**	32	1.921	0.529-6.981	0.321
Number of biopsy cores with PCa: 1 vs. >1*	32	1.756	0.622-4.956	0.288
Maximum PCa infiltration rate: <10 vs. ≥10 %**	32	1.788	0.561-5.702	0.326
Ki-67 positive PCa cells: yes vs. no	32	0.710	0.205-2.464	0.590

* Information from the time of initial diagnosis
** Information from the time of in-house tissue sampling

Time interval in-house tissue sampling until progression

Variable	n	Hazard ratio	95% confidence interval	p
Age: < 65 vs. ≥ 65 years*	32	2.145	0.731-6.296	0.165
PSA: <4 vs. ≥4 ng/ml*	32	0.983	0.795-1.215	0.871
Clinical Stage: cT1c vs. cT2*/**	32	1.120	1.018-1.233	0.020
Number of biopsy cores with PCa: 1 vs. >1*	32	1.737	0.658-4.587	0.265
Maximum PCa infiltration rate: <10 vs. ≥10 %**	32	2.237	0.747-6.698	0.150
Ki-67 positive PCa cells: yes vs. no	32	0.861	0.269-2.761	0.802

* Information from the time of initial diagnosis

** Information from the time of in-house tissue sampling

Supplementary Table S3. Multivariate Cox regression analysis of patients in IHC cohort including a cut-off Ki-67 positivity rate of 5%.

Time interval first diagnosis until progression

Variable	n	Hazard ratio	95% confidence interval	p
Age: < 65 vs. ≥ 65 years*	32	2.298	0.754-7.010	0.144
PSA: <4 vs. ≥4 ng/ml*	32	1.032	0.821-1.298	0.788
Clinical Stage: cT1c vs. cT2*/**	32	1.439	0.423-4.896	0.560
Number of biopsy cores with PCa: 1 vs. >1*	32	1.444	0.473-4.409	0.519
Maximum PCa infiltration rate: <10 vs. ≥10 %**	32	1.698	0.575-5.018	0.338
Ki-67 positive PCa cells: >5% vs. ≤5%**	32	1.563	0.519-4.711	0.427

* Information from the time of initial diagnosis
** Information from the time of in-house tissue sampling

Time interval in-house tissue sampling until progression

Variable	n	Hazard ratio	95% confidence interval	p
Age: < 65 vs. ≥ 65 years*	32	2.196	0.724-6.658	0.165
PSA: <4 vs. ≥4 ng/ml*	32	1.006	0.811-1.247	0.958
Clinical Stage: cT1c vs. cT2*/**	32	0.105	1.004-1.216	0.041
Number of biopsy cores with PCa: 1 vs. >1*	32	1.547	0.564-4.241	0.397
Maximum PCa infiltration rate: <10 vs. ≥10 %	32	2.109	0.813-5.471	0.125
Ki-67 positive PCa cells: >5% vs. ≤5%	32	1.672	0.599-4.665	0.326

* Information from the time of initial diagnosis
** Information from the time of in-house tissue sampling

Supplementary Table S4. Log-rank survival analysis of different models combing Ki-67 status with clinic-pathological parameters

	Category	First diagnosis until progression*	In-house tissue sampling until progression*
Ki-67 negative	1 positive biopsy<br>≥ 1 positive biopsy	p=0.037	p=0.137
Ki-67 positive	1 positive biopsy<br>≥ 1 positive biopsy		
Ki-67 negative	< 65 years<br>≥ 65 years	p=0.038	p=0.056
Ki-67 positive	< 65 years<br>≥ 65 years		
Ki-67 negative	DRU unsuspicious<br>DRU suspicious	p=0.141	p=0.095
Ki-67 positive	DRU unsuspicious<br>DRU suspicious		
Ki-67 negative	infiltration rate < 10%<br>infiltration rate ≥ 10%	n.d.	p=0.442
Ki-67 positive	infiltration rate < 10%<br>infiltration rate ≥ 10%		

Ki-67 negative	PSA < 4 ng/ml		
	PSA ≥ 4 ng/ml	p=0.290	p=0.141
Ki-67 positive	PSA < 4 ng/ml		
	PSA ≥ 4 ng/ml		

*Log-rank statistics
